# Monodisperse droplet formation by spontaneous and interaction based mechanisms in partitioned EDGE microfluidic device

**DOI:** 10.1038/s41598-019-44239-7

**Published:** 2019-05-24

**Authors:** S. ten Klooster, S. Sahin, K. Schroën

**Affiliations:** Food Process Engineering, Wageningen University (WUR), Wageningen, The Netherlands

**Keywords:** Fluid dynamics, Applied physics

## Abstract

The partitioned EDGE droplet generation device is known for its’ high monodisperse droplet formation frequencies in two distinct pressure ranges, and an interesting candidate for scale up of microfluidic emulsification devices. In the current study, we test various continuous and dispersed phase properties and device geometries to unravel how the device spontaneously forms small monodisperse droplets (6–18 μm) at low pressures, and larger monodisperse droplets (>28 μm) at elevated pressures. For the small droplets, we show that the continuous phase inflow in the droplet formation unit largely determines droplet formation behaviour and the resulting droplet size and blow-up pressure. This effect was not considered as a factor of significance for spontaneous droplet formation devices that are mostly characterised by capillary numbers in literature. We then show for the first time that the formation of larger droplets is caused by physical interaction between neighbouring droplets, and highly dependent on device geometry. The insights obtained here are an essential step toward industrial emulsification based on microfluidic devices.

## Introduction

Emulsions are mixtures of two immiscible fluids (e.g. water and oil), and form the basis of many products in e.g. the food, pharmaceutical, paint, and cosmetics industry. Features such as the droplet size and its’ distribution determine product quality attributes e.g. texture, appearance and shelf-life to a large extent; i.e. emulsions with a uniform droplet size were shown to be more stable^1–4^. Although emulsions with a droplet size between 0.1–100 μm can be produced at very high production rates of over 20 m^3^ h^−1^^1,5^, controlling droplet sizes is yet far from trivial with classic emulsification techniques, such as high pressure homogenizers and colloid mills^2^. Besides, these technologies typically use only 1–5% of the total energy input to create interfaces, with the remainder dissipating into the system as heat, which is detrimental to heat sensitive components^2^. These technology related disadvantages limit the functionality of the products and their usage in applications where precise droplet sizes are required^4,6^.

In the last two decades, microfluidic emulsification is developing into a promising alternative for the conventional techniques, because of its mildness, energy efficiency and outstanding control over the droplet size (distribution)^7^. As a result, the microfluidic emulsification technique can be used to make products with well-defined specific functionalities, such as: triggered release of flavour or other active components and a reduced caloric load when using double emulsions^8,9^, and the accurate release of medical doses from smart targeted drugs^10^. Besides, there are applications of microfluidic emulsification in chemistry, particle synthesis and biology, where this technology, applied at relatively small scale, was essential for experiments that otherwise would not have been possible^11–13^. Although the current paper focusses on the production of oil-in-water emulsions, which is relevant for e.g. food production, it is good to mention that for biological and biomedicine applications, water-in-oil emulsions are often more relevant. Production of water-in-oil emulsions can be accomplished with microfluidic devices, provided that the microchannels walls are made hydrophobic (for oil-in-water emulsions the devices are hydrophilic) by surface modification or by simply using hydrophobic materials for the production of the devices^14^. It can be challenging to keep the channel walls hydrophobic because emulsifier molecules may adsorb to the surface, leading to wettability changes of the microchannel walls, which can even impede droplet production^15^.

Multiple microfluidic emulsification devices have been introduced in literature and most of them use the shear of the continuous phase to snap-off droplets from the to-be-dispersed phase (e.g. T- and Y-junctions, Flow-focussing and Co-flow devices)^7^. Their working mechanisms are relatively well-understood, and frequencies of several tens of kHz per droplet formation unit (DFU) have been recorded^16^. The maximum production rates of microfluidic emulsification are typically in the order of ml h^−1^ (for droplets <10 μm), which is far too low for industrial scale, and therefore up-scaling of microfluidic devices needs to be explored further. However, for monodisperse droplet formation to take place, the flow rates of both phases need to be controlled accurately at the level of single droplet formation units, making upscaling of these devices rather unpractical^17,18^.

**Figure 1:**
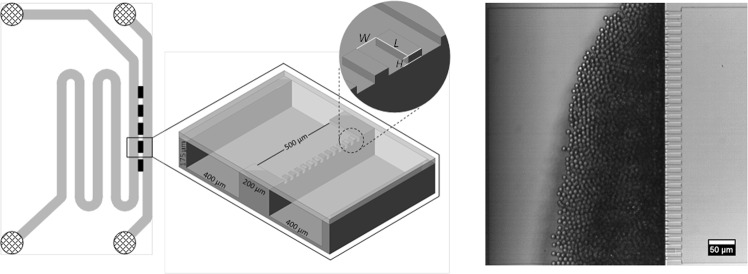
The partitioned EDGE chip layout with five plateaus (black rectangles in left image), placed between the deep continuous and to-be-dispersed phase channels, and a close-up sketch of the micro-plateaus with their characteristic dimensions labelled in the middle circle. On the right: a screenshot of small monodisperse droplet formation.

In spontaneous droplet generating devices, first discovered by T. Kawakatsu *et al*.^19^, interfacial tension forces cause droplet snap-off, and no continuous phase shear is needed. This may facilitate upscaling, although it should be noted that the maximum recorded frequencies (for droplets <10 µm) per DFU are rather low, about 1–1.5 kHz^18,20–22^. Many spontaneous devices with different names and geometries have been introduced in literature (like: Microchannel^19,23^, regular step^22,24^, and facilitated by the co-flowing continuous phase^6,25,26^) and also attempts to up-scale have been undertaken using: (asymmetric) straight-through arrays^27–29^, Millipede^14,30^, microchannel^31,32^, and step emulsification^22^. The spontaneous EDGE device (Edge based Droplet GEneration) developed in our lab, stands out because it can produce multiple droplets from one droplet formation unit connection simultaneously^33,34^. The device consist of a relatively wide, long and shallow slit (the plateau), which connects the to-be-dispersed phase channel to the continuous phase channel (see Fig. 1 for a general impression; please disregard the micro-plateaus for the regular EDGE design). At the position where the plateau and relatively deep continuous phase channel meet, droplets are generated.

When many plateaus are connected to a single feed tube, productivity is improved greatly^35^. To further increase the productivity of regular EDGE, the partitioned EDGE device was introduced, in which the end of the plateau is divided into a number of so-called micro-plateaus (see Fig. 1). This seemingly small geometrical adjustment has shown to increase throughputs by a factor 100 compared to regular EDGE. It was found that at relatively low dispersed phase pressures (<1 bar) these devices produce monodisperse, small droplets (<10 μm), whereas they produce monodisperse, larger droplets (+−30 μm) at elevated pressures (1–3 bar), making the device unique, but also to this date poorly understood^21^.

**Figure 2:**
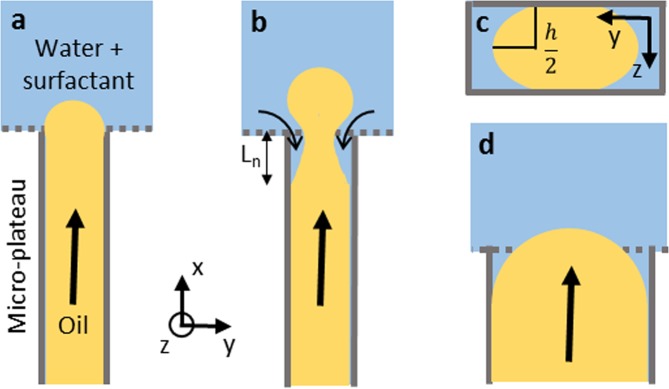
Schematic representation of oil (the to-be-dispersed phase) flowing through a channel that completely confines the liquid, and when the tip reaches the deeper continuous phase channel, it adopts a spherical shape (**a**). When more oil flows into the droplet, this causes the formation of a neck, and subsequently, inflow of continuous phase fluid onto the micro-plateau (**b**). The radius of curvature in the y-z plane is always fixed by the channel height and assumed to be h/2 (**c**). When increasing the micro-plateau width, some continuous phase always resides on the micro-plateau (**d**).

As a starting point, we briefly discuss droplet formation in spontaneous devices^24,36^. When the pressure applied on the to-be-dispersed phase exceeds the Laplace pressure working in the opposite direction, the to-be-dispersed phase starts to flow over the micro-plateau, towards the deeper continuous phase channel^37^. The Laplace pressure is defined as:

1 $${\rm{\Delta }}p=\gamma (\frac{1}{{r}_{1}}+\frac{1}{{r}_{2}})\cos (\theta )$$

where *γ* is the interfacial tension, *r*_1_ and *r*_2_ are the radii of curvature and *θ* is the contact angle between the channel walls and the continuous phase. Arrived at the continuous phase channel, the oil leaps over the edge, where it expands, leading to a droplet-like shape, still connected to the feed (see Fig. 2a,b). In time, more oil flows into the droplet and its radius increases, thereby decreasing the Laplace pressure working on the droplet:

2 $${\rm{\Delta }}{p}_{d}=\frac{2\gamma }{{r}_{d}(t)}$$

where *r*_*d*_ is the radius of the droplet. To equilibrate the pressure between the oil on the micro-plateau and the droplet in the continuous phase channel, the to-be-dispersed phase adopts a negative in-plane curvature (x-y) (see Fig. 2b), because the out-of-plane curvature is fixed by the plateau height (see Fig. 2)^24,33^. This phenomenon causes an under pressure on the micro-plateau, and thereby inflow of the continuous phase, which in turn stretches the neck and decreases the in-plane curvature (see Fig. 2b). Because of the in-, and out-of-plane curvature development at the location of the neck, the local Laplace pressure increases, which eventually causes the neck to collapse and the droplet to be formed^24^. When the applied pressure is increased beyond the so-called blow-up pressure, the device will produce larger droplets of various sizes. Beyond this blow-up pressure, the surface tension force is thought to hardly (or not at all) be able to overcome the viscous force of the to-be-dispersed phase in the neck^38^, which is mostly characterized by a (critical) capillary number: the ratio between viscous and interfacial tension force^39^:

3 $$C{a}^{\ast }=\frac{{\eta }_{d}{U}_{d}^{\ast }}{\gamma }$$

where *η*_*d*_ is the dispersed phase viscosity and *U*_d_^*^ the to-be-dispersed phase velocity at the blow-up pressure. The continuous phase viscosity (*η*_*c*_) is not accounted for in this equation, while we expect that *η*_*c*_ has a significant effect on the stretching of the neck, and thereby on the blow-up pressure (and droplet size), as we will describe later in more detail.

From literature various indications on the (ir)relevance of continuous phase inflow can be found. Recently it was reported that the continuous phase viscosity does not affect the droplet size (mainly) at high viscosity ratios:$\,{\eta }_{d}/{\eta }_{c}$, and that the inflow of the continuous phase is sufficiently slow to be ignored^40^. Others found that the contact angle of the channel walls, which is directly related to continuous phase inflow, has a significant influence on droplet formation^34,41–43^. Also, facilitating in-flow by adding so-called shunt channels has been shown to significantly increase the critical flowrate at which monodisperse droplet formation is lost^44^. Even more specifically, the configuration of the neck is linked to droplet sizes and transition flow rates in analytical models^45,46^. Although often assumed constant, the length of the neck has been shown to decrease upon increasing the to-be-dispersed phase flow rates^24,46^. All of the above indicates that the continuous phase flow around the neck could be rather essential to understand droplet formation, but very little is known to date.

In this paper we focus on the neck dynamics (Fig. 2b), and unravel the droplet formation mechanisms in both monodisperse regimes of the partitioned EDGE device^21^. We find a significant effect of geometry (Fig. 2a,d) on droplet formation behaviour, and of the viscosities of both phases on the blow-up pressure and droplet size. To account for these effects, we derive scaling relations that show excellent agreement with our experimental data obtained for a wide range of experimental conditions.

## Results

The regular EDGE device shows one monodisperse droplet formation regime^33^, whereas the partitioned EDGE device (Fig. 1) has two monodisperse droplet formation regimes^21^.

In the first regime at <1 bar, the device produces small and monodisperse droplets (6–18 μm), at typically 100-fold higher maximum throughput than the regular EDGE device. In the second regime, at 1–3 bar, larger (>28 μm) monodisperse droplets are produced. Both pressure ranges are rather wide compared to other microfluidic devices^21^. To understand the underlying droplet formation mechanisms, we vary the geometry of the device and the continuous and dispersed phase liquids (to obtain a wide range of viscosity ratios: Table 1).

**Table 1:** Viscosities and densities of dispersed and continuous phases in the left and top section, with their respective viscosity ratios (${\boldsymbol{\zeta }}=\frac{{{\boldsymbol{\eta }}}_{{\boldsymbol{d}}}}{{{\boldsymbol{\eta }}}_{{\boldsymbol{c}}}}$) as entries in the table.

	MilliQ η_c_ = 1.0 mPa s ρ_c_ = 997 kg/m^3^	20% Glycerol η_c_ = 1.74^56^ mPa s ρ_c_ = 1040 kg/m^3^	40% Glycerol η_c_ = 3.69^56^ mPa s ρ_c_ = 1088 kg/m^3^	50% Glycerol η_c_ = 6.0^56^ mPa s ρ_c_ = 1113 kg/m^3^
Decane η_d_ = 0.92^57^ mPa s, ρ_d_ = 730 kg/m^3^	0.92	0.53	0.25	0.15
Hexadecane η_d_ = 3.47^57^ mPa s, ρ_d_ = 770 kg/m^3^	3.47	1.99	0.94	0.58
Hexadecane-paraffin η_d_ = 44.5^52–54,58^ mPa s, ρ_d_ = 850 kg/m^3^	44.5	25.57	12.06	7.42
Silicon oil η_d_ = 50 mPa s, ρ_d_ = 960 kg/m^3^	50	28.74	13.55	8.33

### Regime of small droplets

**Figure 3:**
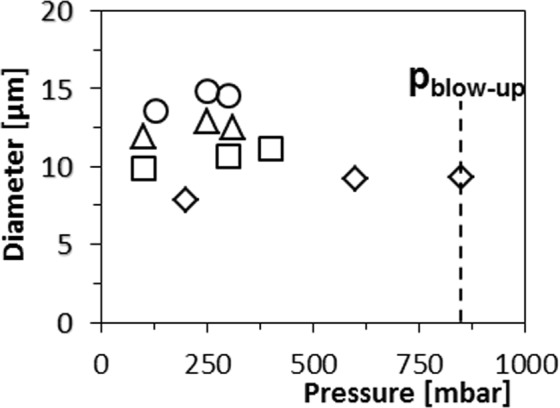
Droplet size generated by PE 5 as function of applied pressure for hexadecane as the dispersed phase and various continuous phase viscosities, going from low to high in the order of **◊**, □, **∆**, ○. Open symbols denote monodisperse droplets with a CV < 10%.

We measure droplet sizes as function of the pressure for various dispersed and continuous phase viscosities with the partitioned EDGE device with the narrowest micro-plateaus (PE 5, width = 5 μm, Fig. 1). As an example, we show the droplet size versus the pressure in the first regime, for hexadecane as the dispersed phase in Fig. 3. Similar graphs obtained for other liquids and device geometries, can be found in the Supplementary Information online: Supplementary Figs 1–3.

**Figure 4:**
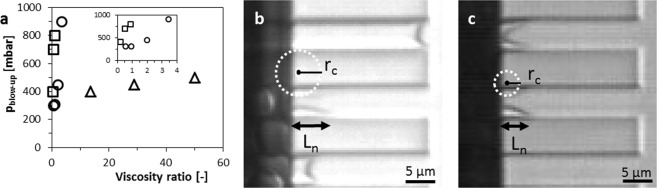
(**a**) The blow-up pressure against the viscosity ratio for PE 5 and for dispersed phases of: silicon oil (∆) (50 mPa s), hexadecane (○) (3.5 mPa s) and decane (□) (0.92 mPa s). (**b**,**c**) Screen shots, in which the black arrow and the dashed circle indicate the length of the neck (L_n_) and the radius of the in-plane curvature (r_c_), respectively at 120 mbar (**b**) and at 900 mbar (**c**) for a hexadecane in water (+0.5 wt.% SDS) system.

For all dispersed and continuous phase fluids, we observe stable monodisperse droplet formation, and the droplet size increases only slightly with pressure. Generally, at higher continuous phase viscosities, the droplet size is larger and the blow-up pressures lower (p_blow-up_) (Figs 3 and 4a). This is in contrast to recent papers in which it is stated that the continuous phase viscosity does not have a significant effect on droplet size, although we need to keep in mind that these authors mainly reported about high viscosity ratios (>10) and somewhat larger droplets (~18  µm)^40^, whereas we test a much wider range as further discussed in the next sections.

Besides the viscosity of the continuous phase, also the accumulation of droplets is thought to influence continuous phase inflow and therewith droplet formation^22,47,48^. We would like to mention that despite a visible accumulation of droplets in front of the droplet formation units (Fig. 1), we observe very monodisperse droplets (most often CV < 5%), and high droplet formation frequencies (of above 1 kHz.). Since we did not investigate accumulation in detail, we cannot confirm that this did not play a role, but expect chances to be low.

### Droplet formation mechanism in the narrowest micro-plateau

As mentioned in the introduction, the driving force for droplet formation in spontaneous devices is the gradient of curvature and the consequent inflow of the continuous phase. This inflow stretches the neck, and in turn the interfacial tension force can trigger an instability, which snaps-off the droplet^24,36^.

The critical capillary number (equation 3) is often taken as the dimensionless number characterizing blow-up^39^; however, it is possibly too simple to characterise the partitioned EDGE device, because: (1) it does not contain a term for the continuous phase viscosity, while continuous phase backflow is speculated to be important for droplet formation^24,36^; (2) often equilibrium interfacial tension is assumed, while dynamic interfacial tensions could play a role^45^; and (3) next to the viscous forces, also the inertial forces could hinder droplet breakup^22^. We will discuss this further below.

We observe a decreasing blow-up pressure with continuous phase viscosity (Fig. 4a), which suggests that the critical capillary number, is not sufficient to capture the behaviour of partitioned EDGE devices. Given the high droplet formation frequencies recently reported for different devices^20–22^, it may be expected that dynamic interfacial tension effects play a role^49^. When comparing with expansion rates obtained in a shear-based system with which dynamic interfacial tension effects were measured^50^, we find that our device is high in Peclet number, defined as:

4 $$Pe=\frac{{v}_{c}h}{D}$$

where *v*_*c*_ is the velocity of the continuous phase and *D* the diffusion coefficient of SDS. For the lowest recorded velocity in our device (*v*_*c*_ = 0.02 ms^−1^ for hexadecane at p_blow-up_), using *h = 2*∙10^−6^ m and *D* = 7∙10^−10^, we find *Pe* = 60, which indicates that in partitioned EDGE devices, convection dominates the transport of surfactant to the interface, allowing us to follow the approach of Muijlwijk *et al*.^50^ for determining the dynamic interfacial tensions. For the relatively viscous silicon oil, the expansion rates are low (even for pressures close to the blow-up pressure) at ~100 s^−1^, and the equilibrium interfacial tension is an appropriate value to use; however, for decane and hexadecane, the expansion rates are >2000 s^−1^, indicating that the acting interfacial tension could be (almost) equal to the bare oil-water interfacial tension.

It is clear that mistakenly using interfacial tension values will influence the critical values denoted to various dimensionless numbers, which can partly explain the difference in reported values. To clarify if the inertial forces play a role as well, we compare the critical Weber numbers with the critical capillary numbers for the three different dispersed phases, in combination with a continuous phase of water with 0.5 wt.% SDS. The critical capillary number is defined in the introduction (equation 3); the Weber number relates inertial and surface tension forces:

5 $$W{e}^{\ast }=\frac{{\rho }_{d}{({U}_{d}^{\ast })}^{2}{d}^{\ast }}{\gamma }$$

where *ρ*_*d*_ is the dispersed phase density and d^*^ the droplet diameter at the blow-up pressure. The critical capillary and critical Weber numbers, based on dynamic interfacial tension values, can be found in Supplementary Table 1. In that table it is shown that the critical Weber number becomes pronounced at high to-be-dispersed phase flow velocities, for fluids with a low viscosity, indicating that inertial forces could hinder break-up of the neck. When looking at the critical capillary number it is clear it increases with the viscosity of the dispersed phase, which is expected to be a result of low inertial forces for high dispersed phase viscosities at blow-up. This confirms that the inertial force of the to-be-dispersed phase, cannot be neglected at blow-up for low dispersed phase viscosities (in line with another recent study)^22^, and could possibly hinder break-up of the neck.

When zooming-in, we generally see that the length of the neck (*L*_*n*_) decreases and the in-plane curvature increases $({\kappa }_{c}=1/{r}_{c})$, with the applied pressure (see Fig. 4b,c), which is in line with other studies^24,46^. The increase in *κ*_*c*_ causes a decrease of the total Laplace pressure (equation 1), because the in- and out-of-plane curvatures point in different directions. This is unfavourable for droplet snap-off, because the local Laplace pressure (equation 1) in the neck decreases, whereas a high local Laplace pressure is needed to impose the instability. To link this phenomenon to its consequences, we vary the continuous phase viscosity, to influence the continuous phase inflow, and therewith *κ*_*c*_. We hypothesize that increasing the continuous phase viscosity by a factor 2, decreases the ‘inflow rate’ by a factor 2 using Hagen-Poiseuille ($Q \sim 1/\eta$, where *Q* is the flow rate [in ${m}^{3}/s$]). The time needed to stretch the neck by the continuous phase inflow, and trigger the instability is thus expected to become twice as long if the continuous phase inflow is decreased by a factor two, and the droplet volume will be doubled as well. In other words:

6 $$\frac{{\eta }_{c}}{{\eta }_{c,ref}}\approx \frac{{V}_{drop}}{{V}_{drop,ref}}$$

where *η*_*c*_ is continuous phase viscosity, *η*_*c*,*ref*_ the reference continuous phase viscosity, *V*_*drop*_ the volume of the droplet produced in a continuous phase with viscosity *η*_*c*_ and *V*_*drop*,*ref*_ the volume of the reference droplet. When rewriting to droplet sizes (*d*) instead of droplet volumes we expect that:

7 $$\frac{d}{{d}_{ref}}\approx {(\frac{{\eta }_{c}}{{\eta }_{c,ref}})}^{\frac{1}{3}}$$

**Figure 5:**
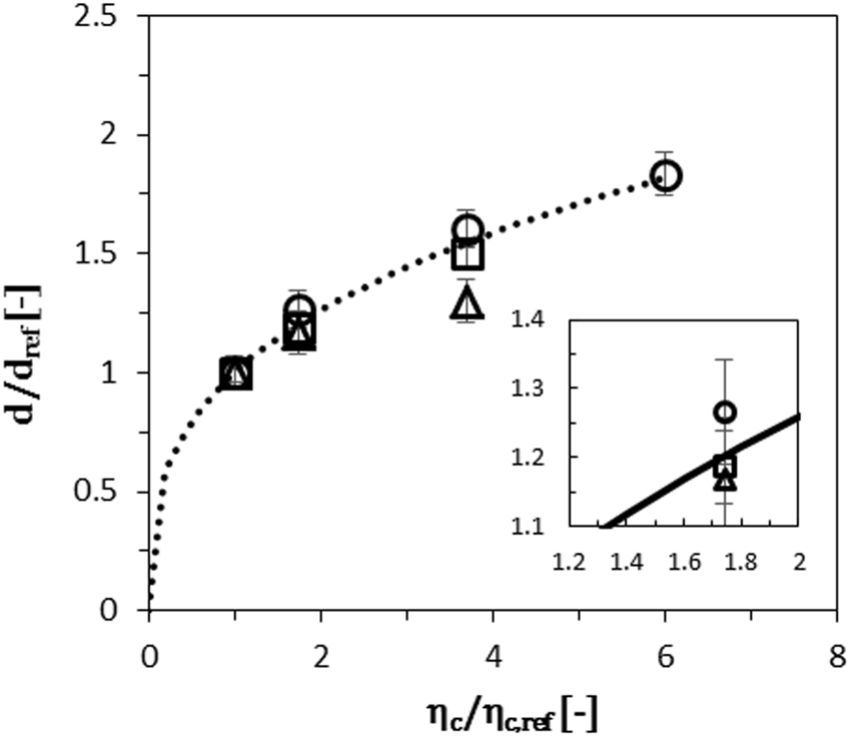
The normalised droplet size (d/d_ref_), versus the normalised viscosity of the continuous phase (η_c_/η_ref_) for PE 5 devices, and dispersed phases of silicon oil (**∆**) (50 mPa s), hexadecane (**○**) (3.5 mPa s) and decane (**□**) (0.92 mPa s). Dashed line indicates the theory as described in the text.

We take the droplet sizes (normalised for pressure, see: Supplementary Information) produced in a continuous phase of MilliQ + 0.5 wt.% SDS as a reference, and plot $(d/{d}_{ref})$ against $({\eta }_{c}/{\eta }_{c,ref})$, for different dispersed phase fluids. We find a good agreement with data for decane (**□**) and hexadecane (**○**) (see Fig. 5). This result confirms the importance of the inflow of the continuous phase around the neck for triggering the instability in narrow micro-plateaus. For silicon oil (∆) (relatively high viscosity), the theory slightly overpredicts the data (Fig. 5), as explained later.

Next, we focus on the configuration of the neck. The inflow of the continuous phase is caused by an under pressure, created by the gradients of curvature of the neck^24^. However, the capillary pressure also drives the inflow of the continuous phase^51^:

8 $${p}_{Ca}=\frac{2\gamma \,\cos (\theta )}{r}$$

with *r* the radius of a round capillary. The velocity can be estimated with a simplified version of the Lucas-Washburn equation^51^:

9 $${U}_{c}=\frac{\gamma \,r\,\cos (\theta )}{4{\eta }_{c}s}$$

with *s* denoting the distance travelled by the fluid, which is in our case the length of the neck (*L*_*n*_) and *r* the radius of the cross-sectional area (approximated as round for simplicity), through which the continuous phase flows onto the micro-plateau. Taking some representative values (see: Supplementary Information), equation 9 shows us that *U*_*c*_ could be in the order of 1 ms^−1^. The corresponding Reynolds number for continuous phase inflow can now be calculated:

10 $$Re=\frac{{{\rm{\rho }}}_{c}{U}_{c}D}{{\eta }_{c}}$$

where *D* is the characteristic length scale for which we take the height of the micro-plateau (2 μm) and for *U*_*c*_ the value from equation 9. Filling in the density and viscosity of water, gives us $Re\approx 1$, which indicates that the inertial force of the continuous phase inflow cannot be neglected.

For silicon oil to reach the velocities calculated with equation 9, the to-be-dispersed phase has to withdraw fast enough (i.e. form a neck on the micro-plateau), and for the very viscous silicon oil, this is probably the limiting factor. Increasing the continuous phase viscosity will not largely affect the continuous phase inflow velocity due to the high viscous force in the to-be-dispersed phase, even at low flow velocities. Therefore, the continuous phase inflow slightly affects the blow-up pressure, which is in line with van Rijn *et al*.^46^, who showed that at moderate Ca/Ca^*^, the more viscous to-be-dispersed phase has the shortest neck, suggesting that it is least stretched.

**Figure 6:**
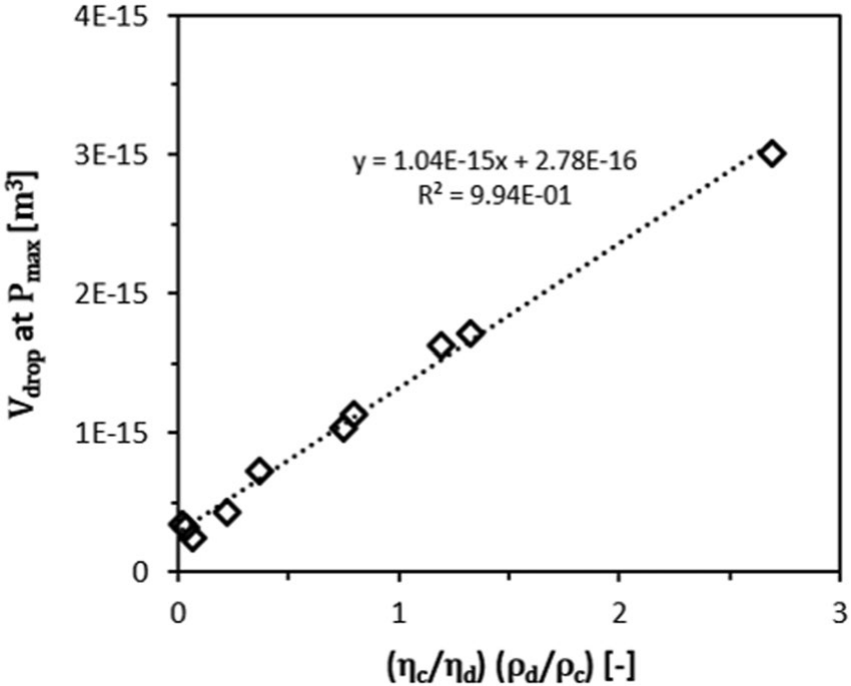
The linear relationship of droplet volume (V_drop_) just below the blow-up pressure (p_blow-up_) and the scaling explained in the text (η_c_/η_d_) (ρ_d_/ρ_c_) for partitioned EDGE devices with a micro-plateau width of 5 μm.

This leads to the general conclusion that the inertial forces of both phases need to be taken into account, and that the droplet volumes produced at pressures just below the blow-up pressure, would need to scale with the densities of both fluids, next to their viscosities as previously found^34^. Here we use $({{\rm{\rho }}}_{d}/{{\rm{\rho }}}_{c})/({\eta }_{c}/{\eta }_{d})$ at blow-up pressure: *ρ*_*d*_ increases the inertial force, which discourages droplet break-up, *ρ*_*c*_ increases continuous phase inertial force, leading to smaller droplets; *η*_*c*_ decreases continuous phase inflow rate, and *η*_*d*_ reduces the flow rate at which blow-up occurs. When plotting the volume of the droplets just before the blow-up pressure is reached against $({{\rm{\rho }}}_{d}/{{\rm{\rho }}}_{c})/({\eta }_{c}/{\eta }_{d})$ we find a linear relationship (see Fig. 6), confirming that all factors play a role.

### Droplet formation mechanism in wider micro-plateaus

Similarly to the PE devices with narrow micro-plateaus, for the wider versions (20 and 40 micrometer), we see that the droplet size and blow-up pressures are dependent on the viscosity of the continuous phase at moderate and low viscosity ratios (<10) (Supplementary Figs 2, 3 and 5), indicating that also for these devices the critical capillary number fails to characterise droplet formation. At high viscosity ratios (>10), the droplet sizes and blow-up pressures become totally independent of the viscosity ratio, which is in contrast with the narrow micro-plateaus discussed before.

**Figure 7:**
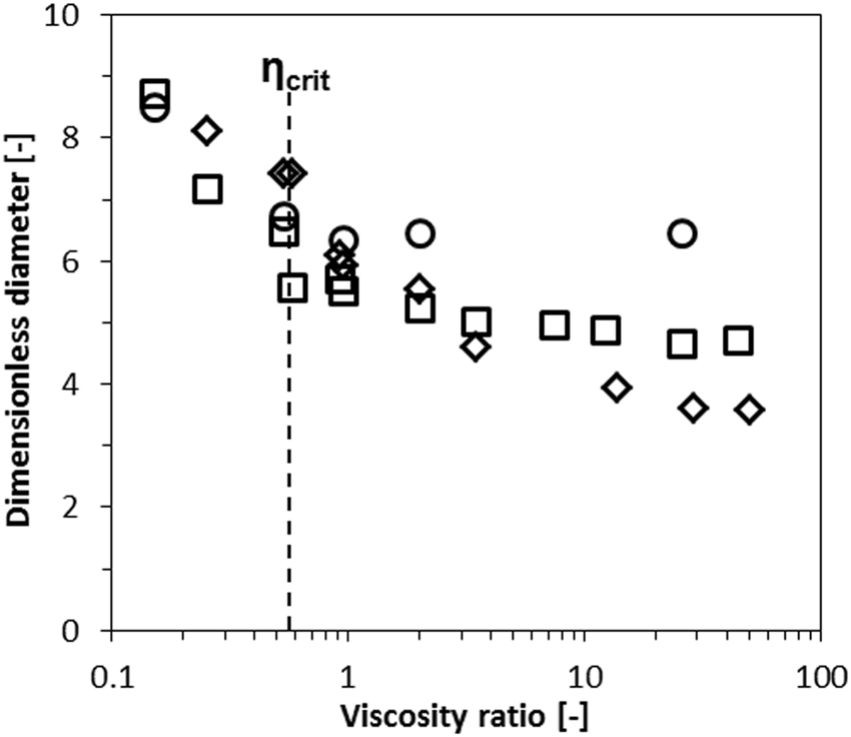
Effect of the viscosity ratio on the droplet diameter for partitioned EDGE devices with a micro-plateau width of 5 μm (**◊**), 20 μm (**□**) and 40 μm (**○**).

To compare all devices, we use a plot of dimensionless droplet diameter (droplet diameter at low pressure/plateau height) versus the viscosity ratio (Fig. 7). Especially for the wider micro-plateaus, the droplet size is found to be constant at high viscosity ratios, whereas larger droplets are found at lower viscosity ratios. This is in line with findings of van Dijke *et al*.^49^ for microchannel emulsification and regular EDGE^18,34^. The critical viscosity ratio (*ζ*_*crit*_) is around 0.6 for both wider microplataus (viscosity ratio at which the droplet size becomes ‘constant’), and is similar for other devices^28,35,41,43–45^.

The narrowest micro-plateau does not really show such inclination point. At high viscosity ratio, the narrow micro-plateaus produce the smallest droplets, but at low viscosity ratios, the droplets produced by the wider micro-plateaus are smaller (Fig. 7). In the wider micro-plateaus the continuous phase inflow is less constricted, and as a consequence, equation 7 does not hold anymore (Supplementary Fig. 6), leading to relatively smaller droplets.

### Stable formation of large monodisperse droplets

Generally, the droplet sizes in spontaneous microfluidic emulsification devices increase slightly with applied pressure, but become large and polydisperse at values above the so-called blow-up pressure^34^. The related uncontrolled growth in droplet size is caused by an insufficiently high local Laplace pressure in the neck. However, droplet snap-off can still be induced, using another driving force to impose an instability (for example buoyancy)^40^.

In contrast with most spontaneous microfluidic emulsification devices, the larger droplets produced by the narrowest partitioned EDGE devices are often still monodisperse (Supplementary Figs 1 and 2), with droplet sizes that are largely independent of the viscosity ratio (Supplementary Fig. 7) and applied pressure (Supplementary Figs 1 and 2). The droplets produced are about 28 μm, which is 14 times larger than the micro-plateau height, and may cause direct contact between droplets forming at neighbouring micro-plateaus. A small calculation shows that neighbouring droplets will be in direct contact when they both have a diameter of 15 μm. When considering the final droplet size (*d* = 28 μm), it takes 5.5 times longer to grow from 15 to 28 μm, compared to from 0 to 15 μm; the forming droplets can be expected to be in direct contact with their neighbours almost all the time.

**Figure 8:**
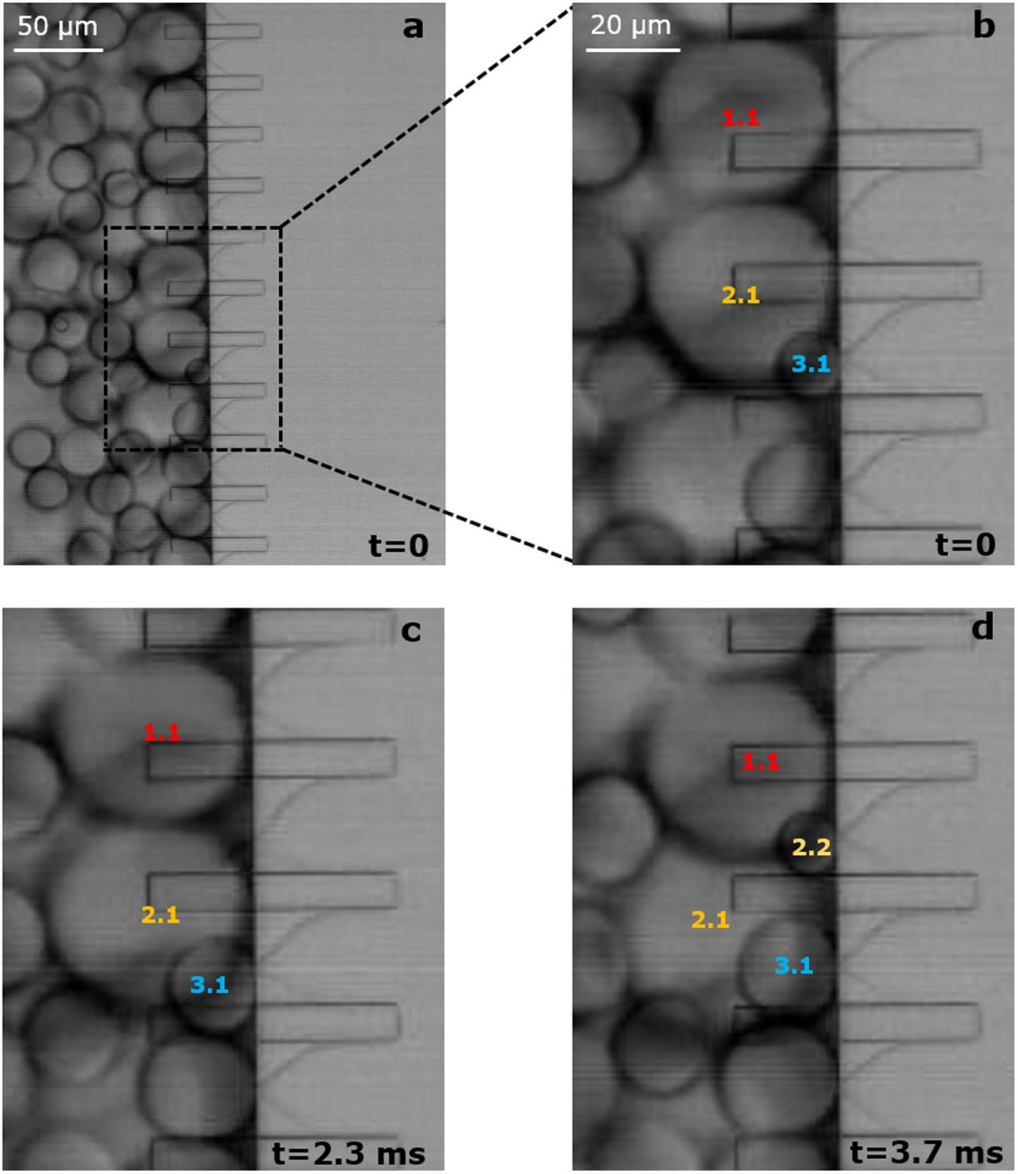
Screen shots showing the droplet formation mechanism in the second monodisperse regime in the partitioned EDGE [20 × 2] device. (**a**) Formation of large and monodisperse droplets. (**b**) Zoomed-in screenshot of four micro-plateaus producing four droplets. (**c**,**d**) Representation of how droplet 2.1 snaps-off due to physical contact with droplet 1.1 and 3.1.

To visualise these effects, we zoom-in on a few micro-plateaus (width = 20 μm) for hexadecane in water with 0.5 wt.% SDS. After its formation, the thickness of the neck increases in time (at a frame rate of 5000 fps), which indicates that the neck is indeed too stable for the interfacial tension force to impose the instability that is needed for droplet snap-off, as also reported previously^55^. We also observe that the forming droplets are continuously touching their neighbours, and this even leads to situations where the droplets are ‘squeezed’ between neighbouring droplets (Fig. 8a,b). We even noted that after the previous droplet is snapped-off, the newly forming droplet pushes the forming droplet of the neighbouring micro-plateau further downstream (Fig. 8c, droplet 3.1 pushes droplet 2.1). Thereby, the liquid thread connecting the forming droplet with the to-be-dispersed phase on the plateau is stretched, which makes it unstable and causes droplet snap-off (Fig. 8c,d, droplet 2.1 snaps-off and droplet 2.2 starts to form, which will in turn impose snap-off of droplet 1.1).

The mechanism underlying this stable droplet formation regime is the physical force that neighbouring droplets exert on each other by direct contact, and as this happens in a cascade fashion (see Supplementary Video 1), this leads to a monodisperse emulsion (only the outer micro-plateaus occasionally form larger droplets due to a lack of neighbouring droplets). This also clarifies why the regular EDGE device and the partitioned EDGE device with the widest micro-plateaus do not show a second droplet formation regime (Supplementary Fig. 3)^33,34^; the droplet forming units are simply too far apart to allow droplets to influence their neighbours snap-off.

Just to be complete, the resulting larger droplets of the micro-plateaus with a width of 20 µm are in general more polydisperse than the larger droplets formed by the micro-plateaus with a width of 5 µm (Supplementary Figs 1 and 2). For these wider micro-plateaus the droplets have to become larger to get into contact and this causes the cascade of events to happen in a less structured fashion than for the narrow micro-plateaus, leading to polydispersity.

To summarise, we unravelled the droplet formation mechanisms for two monodisperse droplet formation regimes in partitioned EDGE devices. The two mechanisms have in common that they rely on the extension of the neck, either through the liquid properties, or by neighbouring droplet interactions. The droplet formation mechanism for the second regime is completely new, and surprising since a cascade of random interactions leads to monodisperse droplets.

## Conclusion

In this paper, we elucidate how the partitioned EDGE device produces monodisperse, small droplets (6–18 μm, at pressures <1 bar) and monodisperse, larger droplets (>28 μm, at pressures 1–3 bar). Both droplet formation mechanisms in narrow micro-plateaus rely on the extension of the neck, either by continuous phase inflow, or by droplet interactions. The behaviour of the narrowest micro-plateaus can be scaled, using the viscosities and densities of both liquids. For the wider micro-plateaus, the droplet formation mechanism is similar to that described for the regular EDGE device, in which inflow of the continuous phase is much less constricted. In the unique second monodisperse droplet formation regime, the neck is stretched by the neighbouring droplets that physically push each other until droplet formation occurs. This happens in a cascaded fashion, and therefore leads to a monodisperse emulsion.

Our findings suggest that, due to their wide pressure ranges, full activation of micro-plateaus and flexibility regarding the liquids that can be used, the partitioned EDGE device is a good candidate for application on larger scale.

## Materials and Methods

### Continuous and dispersed phase fluids

As continuous phases, 0.5 wt.% SDS (Sodium dodecyl sulfate) in MilliQ water and 0.5 wt.% SDS in 20, 40, 50 wt.% glycerol-MilliQ mixtures are used. Hexadecane, decane, silicon oil, and a mixture of hexadecane and paraffin are used as dispersed phases. In this way, the viscosities of the two phases are varied with a factor of 6 for the water phase, and 54 for the oil phase; the corresponding viscosity ratio $(\zeta ={\eta }_{d}/{\eta }_{c})$ varies with a factor of 333, as illustrated in Table 1.

### Microfluidic chip design

The partitioned EDGE microfluidic chips are designed in our lab and produced in glass by deep reactive ion etching (DRIE) (Micronit Microfluidics, Enschede, The Netherlands). The deep disperse and continuous phase channels, and the shallow plateaus are etched into two separate glass substrates, which are later bonded together and diced. The layout of a microchip and dimensions are shown in Fig. 1; the dimensions of the micro-plateaus are specified in Table 2 (including the coding related to the width of the micro-plateaus).

**Table 2:** Characteristic dimensions of the micro-plateaus (i.e. partitions).

Coding used based on micro-plateau width	Number of micro-plateaus per 500 µm [−]	Dimensions of micro-plateaus [*L* × *W* × *H*] [µm]
PE 5	33	30 × 5 × 2
PE 20	17	30 × 20 × 2
PE 40	10	30 × 40 × 2

### Emulsification

During emulsification, the chip holder is placed under a microscope (Axiovert 200 MAT, Carl Zeiss b.v., Sliedrecht, The Netherlands), which is connected to a high speed camera (MotionPro HS-4, IDT Inc., Tallahassee, FL, USA). Thereby, the droplet formation can be observed inline, and recorded for post-processing (see Fig. 1). Both phases are supplied to the chip through PEEK tubing, with outer and inner diameters of 1/16″ and 0.030″, respectively. The flows are controlled through the inlet pressures using a digital pressure controller (Elveflow®, Paris, France). First, the aqueous phase is pressurized into the chip, and the plateaus are wetted by the continuous phase. Then, the oil is pushed into the chip and after the dispersed phase channel is completely filled, this channel outlet is blocked. Thus, the oil is forced to flow continuously over the plateaus, and eventually transform into spherical droplets when leaping into the deeper continuous phase channel. Since partitioned EDGE is a spontaneous droplet formation technique, the flow of the continuous phase is not needed for droplet formation, but it is necessary to prevent overcrowding of the images and move the droplets towards the outlet. Owing to the design of the EDGE devices, the pressure drop inside the channels is about 1 mbar, which is negligible compared to that on the plateau; therefore, we can safely assume that all plateaus operate at the same pressure.

### Droplet size and size distribution

To determine the average droplet size and size distribution, 20–50 droplets per data point are analyzed by image analysis software. The size distribution of the droplets is expressed in the form of coefficient of variation, CV, which is defined as:

11 $${\rm{CV}}=\frac{\sigma }{{d}_{dr}}\times 100$$

where *σ* is the standard deviation of the droplet diameters and *d*_*dr*_ is the number-average droplet diameter. Droplets with a CV below 10% are considered monodisperse.

### Expansion rate and acting interfacial tension

**Figure 9:**
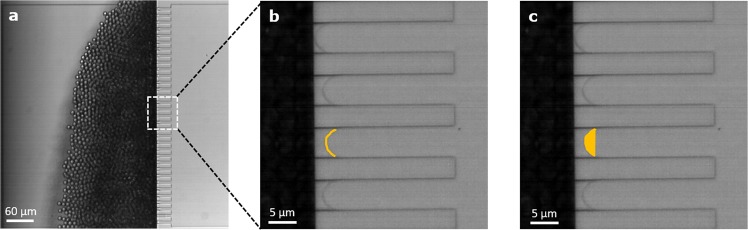
Screenshot of the partitioned EDGE chip when producing small monodisperse droplets (**a**). Zoomed in screenshots of four micro-plateaus with the oil-water interface still on the micro-plateau just before droplet formation, and representation of the determination of the Circumference (*C*) (**b**) and top area (*A*_*top*_) (**c**) at the start of droplet formation.

Recently, the acting interfacial tension during droplet formation was reported for a shear-based system^50^. Following this paper, we calculate the expansion rates and corresponding acting interfacial tensions in a similar way:

12 $$\omega =\frac{ln\frac{{A}_{end}}{{A}_{start}}}{\Delta t}$$

where Δ*t* is the droplet formation time, *A*_*end*_ the surface area of the final droplet and *A*_*start*_ the surface area of the to-be-dispersed phase at the start of droplet formation:

13 $${A}_{start}=C\,h+2{A}_{top}$$

where *A*_*top*_ is the surface when viewed from the top, and *C* the circumference, which are determined at the start of droplet formation with ImageJ (version 1.51 f) (see Fig. 9b,c), and *h* is the micro-plateau height. *A*_*end*_ is calculated using the final droplet size:

14 $${A}_{end}=4\pi {r}_{d}^{2}$$

## Supplementary information

The droplet formation of large monodisperse droplets.

Supplementary information

## Data Availability

The datasets generated during the current study are available from the corresponding author on reasonable request.
