# Supplementary material for: Monodisperse droplet formation by spontaneous and interaction based mechanisms in partitioned EDGE microfluidic device
Source: Sci Rep. 2019 May 24;9:7820. doi: 10.1038/s41598-019-44239-7 (PMC6534564; doi:10.1038/s41598-019-44239-7)
Supplement: Supplementary file 2 — Supplementary information [file 41598_2019_44239_MOESM2_ESM.docx]

Supplementary Information

Monodisperse droplet formation by spontaneous and interaction based mechanisms in partitioned EDGE microfluidic device

S. ten Klooster^a^, S. Sahin^a^, K. Schroën^a^

^a^Food Process Engineering, Wageningen University (WUR), The Netherlands


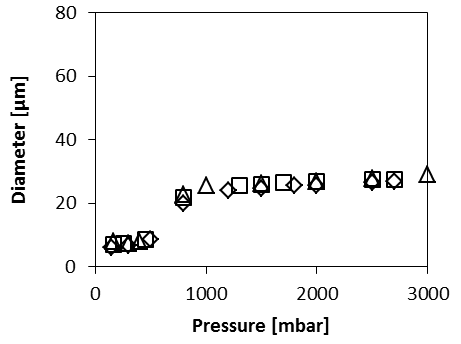

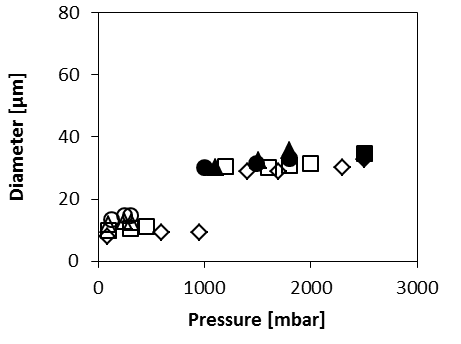

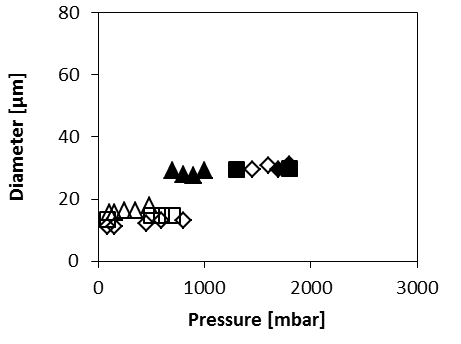


Supplementary Figure 1. Droplet size generated by PE 5 as function of applied pressure for various continuous phase viscosities, going from low to high in the order of ◊, □, ∆, ○. Open symbols denote monodisperse droplets with a CV < 10%, filled symbols denote polydisperse droplets with a CV between 10 and 20%. The used dispersed phases are: (a) silicon oil (η_d_=50 mPa s), (b) hexadecane (η_d_=3.47 mPa s) and (c) decane (η_d_=0.92 mPa s).

**c**

**a**

**b**

Supplementary Figure 3. Droplet size generated by PE 40 as function of applied pressure for: decane/50% glycerol (○), decane/20% glycerol (□), and hexadecane/40% glycerol (∆). All droplets over this pressure range were monodisperse with a CV < 10%.


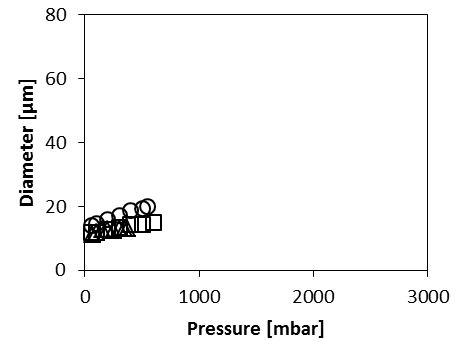

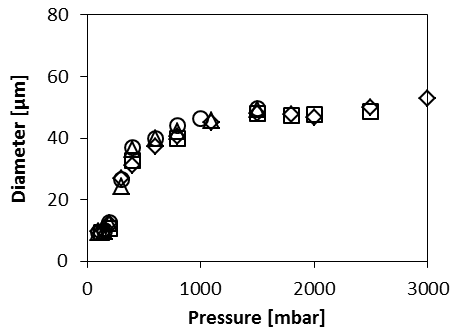

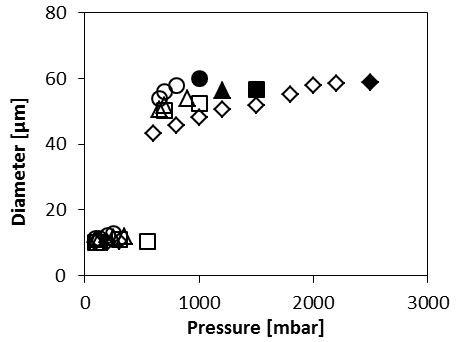

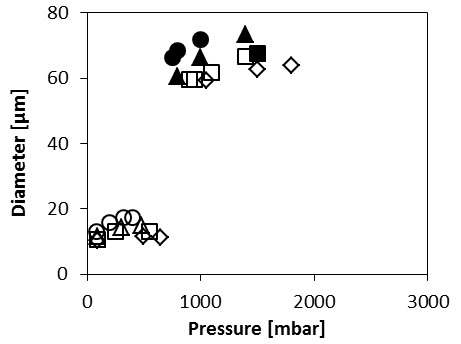


Supplementary Figure 2. Droplet size generated by PE 20 as function of applied pressure for various continuous phase viscosities, going from low to high in the order of ◊, □, ∆, ○. Open symbols denote monodisperse droplets with CV < 10%, filled symbols denote polydisperse droplets with CV between 10 and 20%. The used dispersed phases are: (a) paraffin-hexadecane (η_d_=44.5 mPa s), (b) hexadecane (η_d_=3.47 mPa s), (c) decane (η_d_=0.92 mPa s).

**a**

**b**

**c**

Supplementary Table 1. The expansion rates, (and corresponding) acting interfacial tensions, critical velocity, critical capillary number and critical Weber number for dispersed phases of silicon oil, hexadecane and decane in a continuous phase of water + 0.5 wt% SDS for PE 5.

|  | **ω [s^-1^]** | **γ (mN/m)** | **η [mPa s]** | **ρ [kg/m^3^]** | **d^*^ [m]** | **U_c_^*^ [m/s]** | **Ca^*^ [-]** | **We^*^ [-]** |
| --- | --- | --- | --- | --- | --- | --- | --- | --- |
| Silicon oil | 100 | 7.8 | 50 | 960 | 1.3E-05 | 0.0015 | 9.5E-03 | 3.5E-06 |
| Hexadecane | 2000 | 40 | 3.47 | 770 | 9.3E-06 | 0.040 | 3.5E-03 | 2.9E-04 |
| Decane | 4800 | 52 | 0.92 | 730 | 8.6E-06 | 0.13 | 2.3E-03 | 2.0E-03 |

## Normalizing pressure


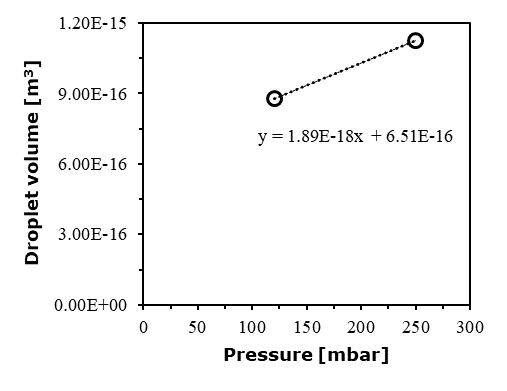


Supplementary Figure 4: Droplet volume against pressure for two data points of hexadecane in water + 0.5 wt.% SDS for PE 5.

The droplet size input, used for figure 6, needed to be normalised, as the pressure has a slight influence on droplet volume. The droplet volumes were linearly interpolated; for example, the droplet size of hexadecane with a continuous phase of 0.5 wt.% SDS and 40 wt.% glycerol was measured at 120mbar and 250 mbar, and needed to be compared at 200 mbar (Supplementary Figure 4). To get a value for the droplet size of hexadecane with a continuous phase of 0.5 wt.% SDS and 40 wt.% glycerol at 200mbar, we interpolated a droplet volume (V_d_), read the corresponding droplet volume and calculated droplet size by: $d=2\left( \frac{V_{d}}{\frac{4}{3} \pi} \right)^{\frac{1}{3}}$.

## Calculation of continuous phase inflow velocity


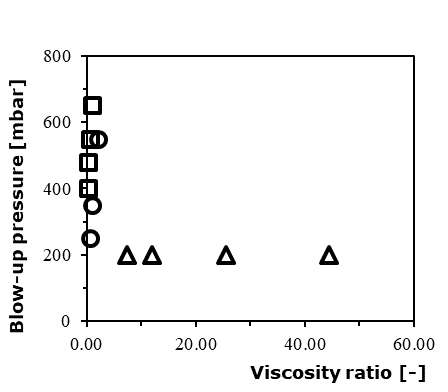

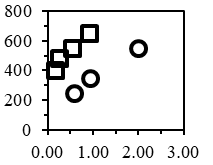


Supplementary Figure 5. The blow-up pressure against the viscosity ratio for: hexadecane-paraffin (∆) (44 mPa s), hexadecane (○) (3.5 mPa s) and decane (□) (0.92 mPa s) for PE 20.

$$U_{c}=\frac{\gamma r cos(\theta)}{4\eta_{c} s}$$

The equation above (for further explanation, see main article), was used to calculate the inflow velocity. For *s* we take a measured distance at which the neck approximately meets the micro-plateau walls (about 4 μm) (see figure 1a), *r* is the diameter of a round capillary of 1.8 μm, the surface is assumed to be fully wetting and therefore 𝜃 = 0ᴼ, *η_c_* =1 mPa s (the viscosity of pure water) and *γ* is taken as the SDS equilibrium interfacial tension (7.8 mN m^-1^). We then find a continuous phase inflow velocity of 0.8 ms^-1^.


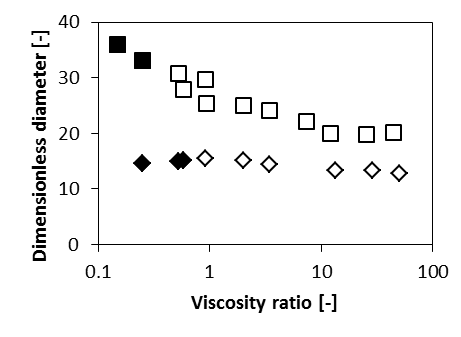


Supplementary Figure 7. Influence of the viscosity ratio on the droplet diameter for PE 5 (◊) and PE 20 (□) in the second monodisperse droplet formation regime (of larger droplets). Open symbols denote monodisperse droplets with a CV < 10%, filled symbols denote polydisperse droplets with CV between 10 and 20%.

Supplementary Figure 6. Normalised droplet size (compared to η_c_= 1mPa s) (d/d_ref_) versus viscosity ratio of continuous phase and reference case, for dispersed phases of decane (0.92 mPa s), at an applied pressure of 90 mbar (grey □) and 300 mbar (black □), and hexadecane (3.47 mPa s) at an applied pressure of 300 mbar (○), for PE 20. Black line indicates the theory as derived for PE 5 in the main article.
